# Progress in Surgery

**Published:** 1899-03-25

**Authors:** 


### Progress in Surgery.

### LARYNGOLOGY.

(*Concluded from page* 419.)

**Tuberculosis of the Larynx** (*continued*).—Inasmuch as disturbances of sensation are subjective, too much significance could readily be attached to them if taken alone as a clue to pulmonary tuberculosis, but in regarding them only as evidence of "hysteria" one is apt to miss important clues to pulmonary tuberculosis. Hyperæsthesia was not infrequently associated with anæmia of the soft palate, and it might be stated that unless the subject is wilfully hostile to laryngoscopy, the more pallid the soft palate the greater the tolerance to laryngoscopy. And when in the course of the pulmonary condition the anæmia passed off the tolerance often went with it. Anæmia of the laryngeal mucosa was noted as present in varying degrees in 157 out of 359 cases. At times it was universal; when patchy it was confined to points where the mucosa was more easily exposed to tension, occasioned by a slight œdema in association with the proliferation of subepithelial vessels. It was as frequently met with in men as in women. Hyperæmia was noted in 117 out of 359 cases, and rather more frequently in males. This hyperæmia must be regarded as a feature distinct from, and not amounting to, acute laryngitis. Acute laryngitis did not occur so frequently as one might previously have anticipated. The hyperæmia was often transient, within a few days giving way to pallor; but at times it was most persistent. These phenomena were also attributed to the proliferation of subepithelial vessels. Disturbances in the vocal function were most frequently met with, often transient, not amounting to more than a weakness of voice or loss of tone. The production of voice called for a greater effort, there was a forgetfulness of office, a sluggishness of one or both cords to act, so that there was phonatory waste before voice is produced, and in connection with this required effort might be mentioned that in early tuberculosis, when the speaking voice is weak, the singing voice may be quite clear. Then the singing voice might suddenly go; this causes the patient to seek advice, and the possibility of commencing tuberculosis should not be forgotten. Transient dysphonia was more commonly met with amongst women, and especially young married women, during pregnancy. Not infrequently in cases of so-called "hysterical aphonia" thoracic signs of pulmonary tuberculosis had been subsequently made out, and it was as well to bear in mind the possibility of diagnosing "hysterical aphonia" in the presence of pulmonary tuberculosis. Impaired movement of the vocal cords apart from paralysis was described, and considered to be myopathic in origin, and due to changes noted in the muscular fibres. With regard to transient œdema of the laryngeal mucosa, the contour in a large number of larynges in early pulmonary tuberculosis when compared with that existing in a normal larynx was found to be partially lost, more particularly in the interarytenoid and arytenoid regions along the ventricular bands, and at the base of the epiglottis. A fine crenating or fringing occurs upon the folds of mucous membrane in the interarytenoid space, not sufficient to be called an excrescence, but met with sufficiently often in pulmonary tuberculosis as to be deserving of attention. An early but transient loss of symmetry in the outline of the arytenoid eminences occurred. The ventricles became less patent, and this loss of patency was commonly associated with some œdema of the inner wall of the ventricular band, which was ascertained clinically in endeavouring to pass a curved platinum hoop into the ventricle in search of tubercle bacilli. A slight œdema of this part of the ventricular band, together with an enfeebled action of the compressor sacculi laryngis must effectually retain sputum laden with tubercle bacilli within the ventricle when once it had been lodged there in the act of coughing. Moreover, it is pointed out by Horne that the choking by a cell proliferation of the ducts of the muciparous glands opening into the ventricle, the closure of the ventricle by the œdema secondary to the proliferation of vessels, and the failure of the compressor sacculi laryngis through changes in the muscle fibres to discharge its contents, all combine to deprive the cords of the mucus necessary for their lubrication. The cords lose the semitranslucent mother-o'-pearl sheen, and present an opaque pallor more approaching to dead ivy-white. With insufficient lubrication superficial erosions occurred; this did not amount to ulceration as met with elsewhere in the larynx, inasmuch as the essential element was absent—namely, the lymphatics.

**Laryngeal Affections in Measles.**—Fraenkel[6] records instances of laryngeal ulceration occurring in cases of measles. The preparations showed deep ulceration on the vocal cords and over the arytenoid cartilages, which extended to the perichondrium and cartilage, causing partial necrosis. One preparation showed a funnel-shaped ulcer at the anterior commissure, at the base of which the necrotic thyroid cartilage could be seen and felt. Another preparation showed, in addition to necrotic changes on the posterior pharyngeal wall, necrosis of the mucous membrane over both vocal processes of the arytenoids, with the necrosed cartilage lying adjacent.

These conditions, if recovery ensues, are associated with hoarseness or difficulty in breathing. They form a parallel to the process observed in some cases of typhoid, and is due to invasion of pyogenic microbes from the surface. He observed four cases in one epidemic, and two in another.

**Non-Diphtheritic Membranous Croup.**—Several cases of membranous croup in which diphtheria bacilli could not be discovered have been recorded. Sainsbury[7] reports a case which occurred in the Royal Free Hospital—that of a child age 16 months with marked laryngeal obstruction, no faucial membrane, no albumin. In the course of twelve hours 8,000 units of antitoxin were injected. Tracheotomy had to be performed, and three days later some membrane came through the tube; but this, like two previous careful bacteriological examinations, only revealed the presence of streptococci. The child recovered. There was no history of contagion with diphtheria, no paralytic sequelæ; nor was there any traumatic cause for the non-diphtheritic membranous croup. Lennox Browne records a case in his "Monograph on Diphtheria[8]" in which the organisms were streptococci and staphylococci. He refers to Billing's statement, that the proportion of membranous laryngitis or tracheitis cases which are not truly diphtherial from a bacteriological test is 14 per cent. P. Davidson[9] has exhibited a membranous cast of the trachea and bronchi, with numerous branches, coughed up by a child supposed to be suffering from diphtheria. Part of this, examined microscopically, was found to contain almost a pure cultivation of micrococci. No Loeffler's bacillus was discovered. Dr. Davidson, however, considered the case undoubted diphtheria. There was membrane on the tonsils and in the respiratory tract. Tracheotomy had been performed for dyspnœa. Diphtheria antitoxin had been injected. The patient was shown still wearing a tracheotomy tube. Dr. Davidson remarked that in several of the most marked cases of diphtheria he had seen no Loeffler's bacillus had been found in the membrane examined.

Another case is reported by Worrall,[10] in a child two and a half years of age, who had been ailing for about five days with cough of the characteristic "croup" type, and almost entire loss of voice. A very marked whitish membrane—not of his "washleather" type—was at the back of the throat, implicating the larynx, but not the tonsils and palate. For three days the child did well with moderate feverishness, and then tracheotomy had to be done. The following day the temperature ran up to 108 9 F., the child dying collapsed in the evening. No bacteriological examination was made, but the doctor believed that the affection was malarial.

[6] Biologische Abtheilung des arztlichen Verein, Hamburg, June, 1898; Munchener Med. Woch., 1898. No. 28; Jour. of Laryng., Oct., 1898. [7] Lancet, Oct. 8, 1898. [8] L., p. 181. [9] Brit. Med. Jour., Mar. 13, 1897; Jour. of Laryng., Oct., 1898, [10] Lancet, Oct. 29, 1898.

### GENITO-URINARY SURGERY.

**Septic Infection of the Urinary Tract.**—The discussion at the last annual meeting of the British Medical Association[1] upon this subject is full of interest and practical importance, and as surgeons we have to reconsider our position. What we have for years spoken of as ropy mucus must now be recognised as pus, its characters being due to the alkaline reaction of the urine. Newman classifies septic renal diseases thus: 1. Purulent embolic nephritis by infection from the blood. 2. Purulent interstitial nephritis, ascending infection by lymphatics. 3. Acute septic nephritis without suppuration, of lymphatic origin. 4. Pylonephritis, by direct infection of lymphatics from the renal pelvis. 5. Pyelitis, uncomplicated. 6. Pyonephrosis, with an obstructed ureter. Now a normal healthy mucous membrane is able to resist the invasion of most pathogenic organisms. Conversely, when the resisting power is diminished by the presence of a foreign body, injury, or from residual urine, then infection is liable to occur. The inflammation is always due to micro-organisms, and is usually preceded by some examination of the bladder, being greatly favoured by any retention of urine.

Embolic suppurative nephritis is really pyæmic, and the foci are most marked in the cortex. Of lymphatic invasions we may have a very acute septic infection without suppuration, rapidly ending fatally. The kidney is crowded with micro-organisms, which may perhaps be obtained in pure culture. In other cases the infective process is less acute, so that abscesses form in the cortex of the kidney under the capsule. When infection takes place along the lumina the minute abscesses tend to form in the pyramids after the uriniferous tubules have become blocked by colonies of micrococci. Rovsing is of opinion that infection takes place from the blood much more frequently than was formerly supposed. He thinks that the bacterium coli is of much less importance than the urea-decomposing microbes. Simple bacteriuria has no signs beyond slight turbidity of the urine. Urea-decomposing non-pyogenic microbes cause a catarrhal cystitis. Pyogenic organisms produce suppurative cystitis. It is now known that there may be no cystitis accompanying primary pyelitis, although the bladder may have been the reservoir of pus for years. Rovsing finds that primary pyelitis is usually due to the bacillus coli, whilst primary cystitis is due to urea-decomposing microbes.

**Air Inflation of the Bladder.**—This was suggested originally by Dr. A. T. Bristow preparatory to suprapubic cystotomy. It is best to cut down on to the transversalis fascia, and then have the bladder distended. Dr. W. Jepson[2] confirms the opinion that air distension raises the peritoneum more completely than water distension by virtue of the fact that it is so much lighter than water. Moreover, the compressibility of air diminishes the risk of rupture of the bladder. A double acting bicycle pump has been used, but Jepson recommends a simple apparatus as follows: Two bottles are provided on adjustable stands. The higher one contains water, which is conducted to a lower bottle containing air, from the top of which a tube passes to the catheter. The water passing from the first bottle into the second displaces the air into the bladder, the pressure of which can be estimated by a small manometer. The air in the bottle and in the bladder are continuous, so that rupture is less likely to occur, for any increased vesical tension expends itself upon the column of water in the bottle.

**Vesical Calculus.**—Mr. Reginald Harrison has recorded a seven years' experience including over 100 operations for this condition. His cases of litholapaxy gave a mortality of 6 per cent. The risk is much enhanced if a stricture have existed on account of associated suppurative nephritis, and in this condition Harrison is of opinion that perineal lithotrity and drainage is the safer method of treatment.[3] He lays stress on the importance of watching old patients with enlarged prostates for some months after operation, as they are so prone to recurrence of calculi.

Harrison speaks favourably of perineal lithotrity in old and feeble subjects, and in those with marked cystitis and probable suppurative nephritis. He thinks sounding should be done under an anæsthetic, and that the surgeon should be prepared to operate straight away.

**Cystoscopy and Ureteral Catheterisation.**—These are now well known methods of diagnosis. Dr. H. N. Vineberg (New York) praises Kelly's urethral specula in the local treatment of vesical states, which he thinks far exceed Nitze's cystoscope in value. David Newman fully realises the diagnostic value of ureteral catheterisation, but thinks it should not be resorted to unless other methods of inquiry have failed to elicit the required information. The fullest possible information should be obtained by the cystoscope, and catheterisation only resorted to when that instrument fails one. Full antiseptic precautions must be taken, and the instruments sterilised. The ureteral catheter should never be passed when the contents of the bladder are septic, lest suppurative nephritis be induced.[4]

Casper, however, from clinical experience, says that the dangers of infection may be regarded as a minimum. He has catheterised the ureters over 500 times, and has never detected any infection.[5] Nevertheless every precaution should be taken, and the ureters only catheterised when it is necessary for diagnosis with a view to treatment. The great value of the examination is to tell which of two kidneys is the diseased one, and if the other kidney be healthy.

[1] Brit. Med. Jour., Oct. 29, 1898. [2] Annals of Surgery, Sept., 1898. [3] Lancet Nov. 12, 1889. [4] Brit. Med. Jour., Nov. 5, 1898. [5] Ditto.

### GENERAL SURGERY.

**Antiseptics.**—A short and valuable summary of the history and value of silver and silver salts in surgery (see The Hospital, September 4th and 18th, 1897) with special relation to wound treatment is recorded by W. S. Bainbridge.[1] He also gives a few examples he had seen at the Carola Hospital under Dr. Credé and the methods employed. The results he considered excellent. The following points were especially noted: No suture irritation or stitch abscesses, no dermatitis, as sometimes seen with iodoform, no systemic effect, even when the amount of the antiseptic was large. It was also found that only a small quantity of the citrate is required. This is important, as the salts are expensive. Solutions must be made fresh, as they are chemically changed if exposed to the light for some time. No corrosion of instruments need be feared, and stains on towels, &c., can be removed by washing, provided they have not been long exposed to the light. Dr. L. V. Lesser, of Leipzic,[2] after using many forms of antiseptics, has decided after three years' experience that the only really good substitute for iodoform is airol (a combination of bismuth, iodine, and gallic acid), in almost all operative and other wounds. He also employs lysol in place of carbolic acid, but still uses the latter for sponges, drainage tubes, sutures, &c., while he sterilises the hands with corrosive sublimate. Orthoform has of late been extensively used in Germany in surgical work. Professor Einhorn and Dr. Heinz[3] have employed it, especially in fresh wounds, burns, ulcers of throat, and carcinomatous and syphilitic ulcers. It is a white powder, and acts as a local anæsthetic where sensory nerve endings are exposed, is non-poisonous and is antiseptic. The excellent results obtained in laboratory work demonstrating the disinfecting powers of the cresols has brought them largely to the front as antiseptic fluids for practical purposes. As Schäffer found that ethylenediamine considerably increased the disinfecting value and penetrating powers of the silver salts, Dr. H. Eckstein[4] wa led to test the combination of a cresol with ethylenediamine. Kremasin is a simple mixture of trikesol with ethylenediamine, has a phenol-like smell, is but slightly toxic, and is miscible in all proportions with water. It has considerable powers of penetrating into organic tissues. Eckstein concludes that kremasin is of the highest antiseptic and disinfectant value, excelling the phenol preparations, that it is non-irritant, and very useful in skin affections, chronic ulcers of the leg, and after treatment of lupus. Mr. Geo. Stoker[5] has found during his treatment of wounds and ulcers by oxygen gas that a distinct toxic reaction occurs in many cases, and that it is followed by more rapid healing. Evidence of this he discovered in the following facts—acceleration of healing after the temperature became highest, the discharge continued healthy, inflammation of lymphatics and glands, formation of secondary abscesses which healed rapidly, and slight general malaise. He concludes that oxygen acts by forming an antitoxin from the secretion of the micro-organisms present in the wound or ulcer, and further suggests the preparation of an antitoxin for each separate case from its own micro-organisms, especially in more malignant forms, it being presumed that an antitoxin must have a toxin present on which to act or react. Opinions still vary considerably regarding the value and possibility of carrying out the aseptic treatment of wounds, what is now fully recognised as a further development of antiseptic principles. Thus Mr. R. Morison, of Newcastle,[6] believes that in this country at least, with the less complete control we have over our surgical work than in other countries, the antiseptic system is simpler, safer, and leads to the most satisfactory results. In this connexion, Mr. C. B. Lockwood[7] gives a continuation of his reports upon aseptic and septic surgical cases. With increased experience they give very uniform results, and the failures seem to be due more to want of thoroughness in the use of the methods employed than to faults in the methods themselves. He concludes that sepsis depends upon a great many causes besides those dealt with in the report, viz., sponges, towels, silk and fishing gut sutures, hands, and patient's skin, and that much remains to be explained. For instance, the skin of the scrotum is exceedingly difficult to disinfect, and, with the exception of the scalp, has a higher proportion of sepsis than any other. Nevertheless, his scrotum wounds did exceedingly well, for he had only one suppuration in 43 wounds made for the cure of chronic hydrocele of the tunica vaginalis. Dührssen,[8] recognising that Lister's theory of the infection of wounds by the access of air had a less significance than the infection by contact, thinks that the principle of asepsis is by no means to be simply or easily realised in a practical manner. He employs an aseptic operating coat, which he removes from the sterilising chamber, having previously washed his hands with a nail brush. In cleansing the hands he brushes them for five minutes in a hot 1 per cent. solution of lysol. The water is almost boiling, so that at first the process is painful. The one disadvantage in using lysol is that it has a pungent smell. Surgeon-General Lühe[9] lately brought forward the subject of asepsis and antiseptics in war. As he considered wounds in war to be generally aseptic, he thought that the aim should be to maintain this condition by protecting the wound from the clothes, the air, and contact with unclean fingers. In the German army there were transportable sterilising apparatus, but there should also be receptacles for the dressings, aprons, &c. An important aim was to provide not only the lazarette reserve depôt, but also the field lazarettes with aseptically prepared material. With the enormous number of wounded to be attended to, the supplies of dressings would soon be exhausted, and could not be sent from home in sufficient quantities; other raw material, peat, shavings, charpie, and the like, must be disinfected and made ready. The sterilising apparatus was under the superintendence of the surgeon-in-chief and his assistant.

[1] N.Y. Medical Record, Oct. 15, 1898, p. 548. [2] Therapist, Feb. 15, 1899, p. 45, and Deutsche Med. Woch, No. 1, 1899. [3] Therapist, July 15, 1898, p. 161. [4] Therapist, July 15, 1898, p. 162. [5] Lancet, Dec. 10, 1898, p. 1,545. [6] Brit. Med. Jour., Nov. 12, 1898, p. 1,481. [7] Brit. Med. Jour., Sept. 17, 1898, p. 802. [8] Berl. Klin. Woch, July 4, 1898, and Epit. Brit. Med. Jour., Aug. 10, 1898. [9] Medical Press, Dec. 21, 1898, p. 650.

